# Different kinds of acupuncture treatments for knee osteoarthritis: a multicentre, randomized controlled trial

**DOI:** 10.1186/s13063-019-4034-8

**Published:** 2020-03-14

**Authors:** Qifei Zhang, Jianqiao Fang, Lifang Chen, Jiayao Wu, Jing Ni, Fang Liu, Jing Sun

**Affiliations:** 1 The Third Clinical Medical College, Zhejiang Chinese Medical University, No.548 Binwen Rd, Binjiang District, Hangzhou Zhejiang China; 2 Department of Acupuncture, The Third Affiliated Hospital of Zhejiang Chinese Medical University, No.548 Binwen Rd, Binjiang District, Hangzhou Zhejiang China; 3 Department of Acupuncture, Third Affiliated Hospital of Zhejiang Traditional Chinese Medical University, NO.219 Moganshan Road, Xihu District, Zhejiang China; 4 Department of Acupuncture, Hangzhou Red Cross Hospital, NO.208 Huanchengdong Road, Hangzhou, Zhejiang China

**Keywords:** Acupuncture, Knee osteoarthritis, Electro-acupuncture, Warm-needling, Sham acupuncture, Mild moxibustion, Multicentre randomized controlled trial

## Abstract

**Introduction:**

Knee osteoarthritis (KOA) is a chronic disease with symptoms of persistent pain or resting pain, joint stiffness, numbness, limitation of activity and even disability, with significant associated costs and effects on individuals’ life quality. The use of acupuncture for the management of chronic pain is receiving increasing recognition from both the public and professionals. The aim of this study is to identify the effects of three commonly used acupuncture treatments for KOA.

**Methods/analysis:**

In a prospective trial involving six hospitals in Zhejiang Province (China), 360 patients with KOA will be included. Eligible patients will be randomized into six groups: Acupuncture, Electro-acupuncture, Mild moxibustion, Warm-needling, Sham acupuncture and Celebrex treatment. Twelve treatment sessions will be performed over a 4-week period. The primary outcome will be the visual analogue scale and Western Ontario and McMaster Universities Osteoarthritis Index (WOMAC) function scores (the average of the past 3 days) at weeks 2 and 4 and at 3-month and 6-month follow-up. Secondary outcome measures will be as follows: the WOMAC pain score and WOMAC stiffness score (the average of the past 3 days); the Physical Activity Scale of the Elderly (PASE); knee joint swelling measurement; the WHO Quality Of Life-BREF (WHOQOL-BREF) life quality scale; and the incidence of adverse events.

**Trial registration:**

ClinicalTrials.gov, NCT03563690. Registered on 2rd July 2018.

## Article summary

This is a single-blinded, multicentre, randomized controlled trial. This trial is expected to clarify whether acupuncture and moxibustion treatment play a key role in the treatment of knee osteoarthritis (KOA). The trial will also assess the efficacy of different kinds of acupuncture treatments and determine the best therapy for KOA under strict quality control.

## Strengths and limitations of this study

- This is one of few scientific and rigid clinical studies that include an effective Western medicine group and a sham needling group at the same time as therapeutic groups.
- The inclusion of six centres in different districts can minimize patient bias between centres.
- This trial is expected to assess the immediate and long-term effects of different kinds of acupuncture treatments as well as the comfort effect of needling in knee osteoarthritis treatment.
- In this trial, blinding will be difficult for most patients who have had acupuncture treatment before; therefore, when the study group undergoes the trial, we will perform each treatment separately for each patient in different rooms in the clinic.

## Introduction

Knee osteoarthritis (KOA) has been recognized as the most common chronic disease affecting elderly people. According to the World Health Organization (WHO), approximately 10% of people over 60 years of age in the world have KOA-related syndromes [1], while in China, the proportion has increased to 19.4% [2]. As the population ages, OA will become the fourth most disabling disease in the world by 2020 [3].

At present, both drug and non-drug treatments are used to treat knee osteoarthritis, and the guidelines mainly recommend non-steroidal anti-inflammatory drugs (e.g., celecoxib, acetaminophen), glucosamine and chondroitin sulfate [4–7]. Although these drugs can relieve pain, they have some limitations; for example, acetaminophen is limited by a weak pain-relief effect, and dose increases are associated with side effects such as liver damage and gastrointestinal tract toxicity [8, 9]. Therefore, drug treatment is not the best choice for KOA, especially for elderly patients.

In recent years, acupuncture has become a popular non-drug therapy with a good curative effect, convenient application, non-toxic side effects and low cost, and it has been widely used in the treatment of KOA. The efficacy of acupuncture and moxibustion treatment for KOA has been well reported in repeated randomized controlled studies, and the curative effect of acupuncture treatment on KOA is also becoming gradually known [10, 11].

However, the efficacy of acupuncture treatment for KOA is not accepted worldwide [12, 13]. The results of these studies have been criticized due to a lack of definitive evidence regarding the efficacy of acupuncture; some studies suggest that acupuncture has clear benefits, whereas others are less conclusive [14]. Many years of clinical practice experience and clinical research by this research group show that the pain of KOA may be associated with chronic inflammatory pain, and moxibustion and electro-acupuncture have a good effect on chronic inflammatory pain [15–17]. At present, no accepted research has confirmed the effectiveness of moxibustion and electro-acupuncture in the treatment of chronic inflammatory pain. Moxibustion can regulate the expression of cytokines, which are closely related to the inflammatory response and play an anti-inflammatory role. It can also relieve KOA symptoms by regulating the gene conduction pathway, activating endogenous opioid peptides and regulating the signal release of the central nervous system [18]. Multidimensional evidence regarding the use of different acupuncture treatments to treat KOA is needed, and it is necessary to establish scientific and standardized acupuncture treatment for KOA as soon as possible. Therefore, a multicentre RCT will be conducted to address methodological flaws.

## Methods/design

### Patient and public involvement

All members of the study group will attend a series of training sessions before the trial, which will ensure that the personnel involved in this trial fully understand the research protocol and every detail described on the informed consent. The study will recruit participants by advertising in local newspapers and on the Internet, health-related TV programmes, the WeChat Official Account and posters in communities and hospitals. If patients are interested in participating, they will be invited to talk face to face with study coordinators to discuss the study and provide information regarding eligibility criteria. If patients are eligible, they will be invited to participate in a series of KOA assessments after receiving a diagnosis by specialists. Patients will be involved in recruitment and in the conduct of the study. We will keep in touch with the participants from recruitment to 6 months after treatment, and the participants will be able to contact us at any time to get information they want. The burden of the intervention will be assessed by specialists in the study group under the supervision of the Ethics Committee of each hospital. Patient advisers will be thanked in the acknowledgements.

### Study design

**Fig. 1:**
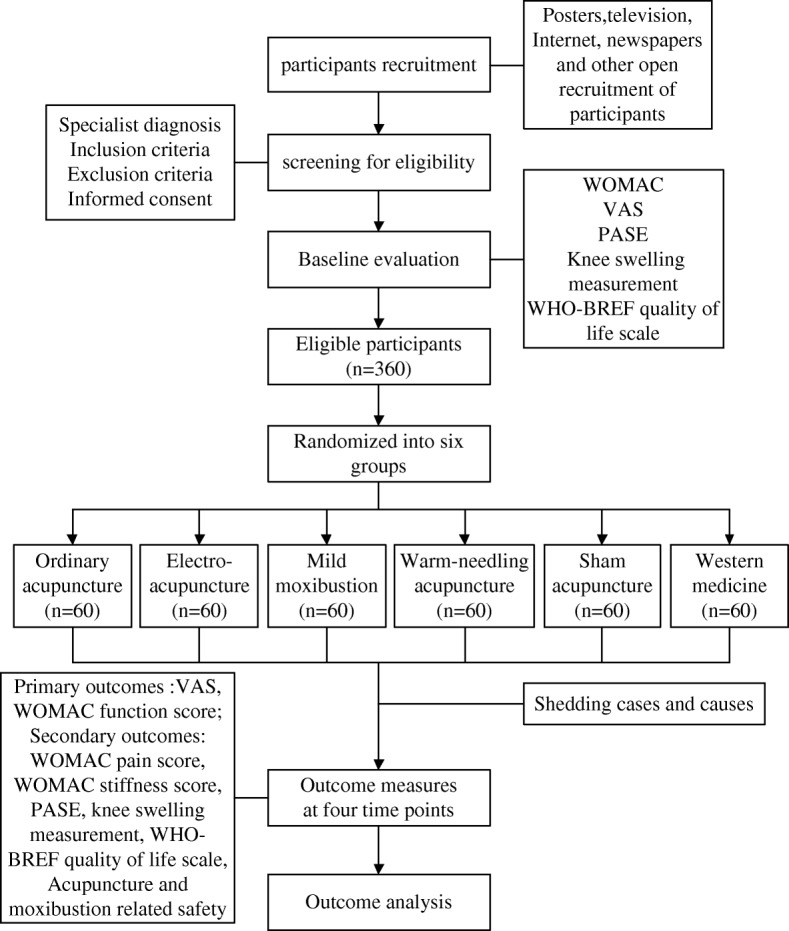
Flow chart of the study process. PASE Physical Activity Scale of the Elderly, VAS visual analogue scale, WHO-BREF WHO Quality Of Life-BREF, WOMAC Western Ontario and McMaster Universities Osteoarthritis Index

This study is a rigorous, randomized, sham-controlled and drug-controlled trial to be carried out in the outpatient acupuncture departments of six different hospitals in Zhejiang Province (China): The Third Affiliated Hospital of Zhejiang Chinese Medical University, Zhejiang Hospital, Zhejiang Provincial Hospital of TCM, Zhejiang Provincial Tongde Hospital, Hangzhou Red Cross Hospital and Jiaxing TCM Hospital. After patients are enrolled and consent is obtained from each of these six clinical centres, 360 participants will be randomized at a ratio of 1:1:1:1:1:1 to six groups (60 participants per group). The participants will receive 4 weeks of individual treatments (one treatment every other day) in six groups: Acupuncture, Electro-acupuncture, Mild moxibustion, Warm-needling, Sham acupuncture and Celebrex treatment. The results will be compared to the effective drug control group. The protocol is based on the Standards for Reporting Interventions in Clinical Trials of Acupuncture (STRICTA) [19] and used the SPIRIT reporting guidelines [20]. The trial design is summarized in Fig. 1.

### Participant recruitment

Participants will be recruited from six first-class hospitals (The Third Affiliated Hospital of Zhejiang Chinese Medical University, Zhejiang Hospital, Zhejiang Provincial Hospital of TCM, Zhejiang Provincial Tongde Hospital, Hangzhou Red Cross Hospital and Jiaxing TCM Hospital) in Zhejiang Province, China. Our study will be advertised in local newspapers and on health-related TV programmes, the Internet and posters in communities and hospitals. Prospective participants will be asked to talk face to face with study coordinators to discuss the study and provide information regarding eligibility criteria. If patients are eligible and interested in participating, they will be invited for a series of KOA assessments after being diagnosed by specialists. Three hundred and sixty consecutive KOA patients admitted to the six hospitals will be included in the study. When informed consent is obtained, the patients will be randomized into six groups with different treatments.

### Inclusion criteria

Patients presenting with all of the following criteria will be considered for study inclusion:

1. Age between 40 and 75 years, male or female
2. Diagnosis of KOA using the diagnostic criteria of the American College of Rheumatology (ACR) [21] and a history of knee pain for more than 3 months and complaints of knee pain on most days of the past month
3. Kellgren–Lawrence (KL) classification of level I–III
4. Average knee pain severity of 4 points or more on a 10-point VAS
5. Voluntary participation in this study and the provision of written informed consent

### Exclusion criteria

Patients with any of the following conditions will be excluded from this study:

1. KOA with a history of gout, infection, tumour, autoimmune diseases, trauma or other causes of knee pain or knee deformities
2. Local skin damage, poor skin conditions or coagulant dysfunction and unsuitability for acupuncture
3. Accompanying serious medical problems or mental disorders, cognitive dysfunction, shallow sensation disorder or inability to cooperate with the treatment
4. History of knee surgery or acupuncture or needle-knife acupuncture within the past month
5. Unsuitability for acupuncture treatment

### Discontinuation criteria and modification

Participants who meet any of the following criteria will be withdrawn from this study.

1. When a patient develops knee pain that cannot be alleviated by acupuncture (based on a VAS score > 8 points), the physician will evaluate the severity and discontinue the study.
2. In the case of the onset of serious adverse events such as severe infection, coma, shock or death, the main investigator will be contacted immediately, and the study will be suspended immediately.
3. The participant decides to discontinue treatment at any time for any reason.

### Ethics and informed consent

The study protocol was reviewed and approved by the ethics committees of the Third Affiliated Hospital of Zhejiang Chinese Medical University, Zhejiang Hospital, Zhejiang Provincial Hospital of TCM, Zhejiang Provincial Tongde Hospital, Hangzhou Red Cross Hospital and Jiaxing TCM Hospital in accordance with the Declaration of Helsinki (ZSLL-KY-2017-030, 2018KA006, 2018-KL-010-01, 2,018,019, 2018 LS05 and 2018-JZLS-001). The protocol was registered at ClinicalTrials.gov (identifier: NCT03563690). The purpose, procedure and potential risks and benefits of the study will be fully explained to the patients and their families by research staff. All patients will give their written informed consent before participating in the study. This trial will not be collecting any biological sample. Participants’ data from this trial will not be used in future studies.

### Randomization and blinding

#### Randomization

The randomization scheme of the study will be generated by the Evaluation and Analysis Centre of Zhejiang Provincial Hospital of TCM. After recruitment, an independent research staff member at each centre who is responsible for randomization will log into the central randomization stochastic system to perform randomization. The central randomization system has strict personnel authority, and other than the highest-level system management staff, no one has the authority to view the randomization scheme in the central randomization system.

#### Blinding

The participants will be blinded to group allocation throughout the study. They will listen to an explanation that they will receive KOA treatment using one of the six interventions with or without acupuncture. The assessor, data recorder, acupuncturist and statistician will all operate independently; the randomization staff and acupuncturist will know the allocation information, while the assessor and statistician will stay blinded to this information throughout the study. Each participant will be treated separately to prevent any exchange of study information. In the case that withdrawal occurs, the study research assistant would provide the relevant information for the participant, which includes the participant’s treatment assignment and outcome data.

## Interventions

**Fig. 2:**
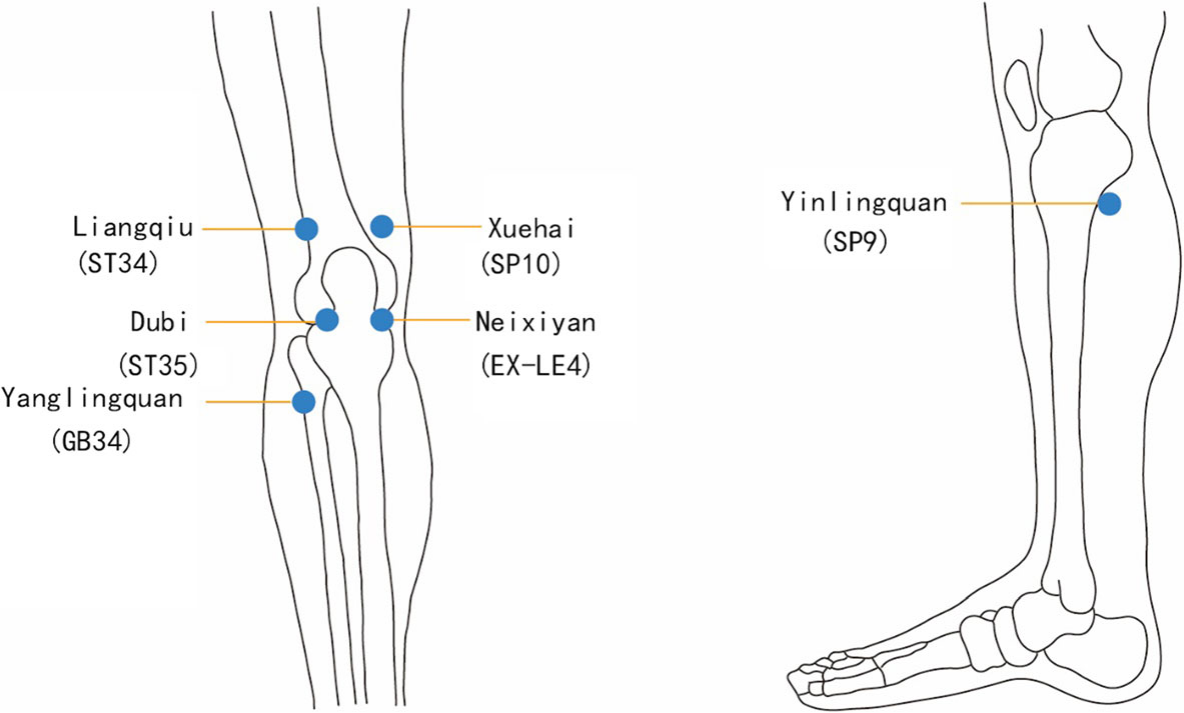
The acupoints used in the study

The patients will be guided to sit with their knees bent naturally at 90° with the affected knee(s) exposed. As shown in Fig. 2, based on TCM theory and a documentary review of previous clinical trials [22–24], the acupoints selected for all but the Sham acupuncture group are Liangqiu (ST34), Xuehai (SP10), Neixiyan (EX-LE4), Dubi (ST35), Yanglingquan (GB 34) and Yinlingquan (SP9).

The unified needle is a disposable sterile acupuncture needle (0.30 mm × 25 mm or 0.30 mm × 40 mm; Huatuo Brand, Suzhou Medical Supplies Factory Co., Ltd, Jiangsu, China). Needles with sizes of 0.30 mm × 25 mm, 0.30 mm × 40 mm and 0.18 mm × 25 mm will be used in the Acupuncture group, the Electro-acupuncture group, the Warm-needling acupuncture group and the Sham acupuncture group.

The unified moxa is Qing Ai 2 cm in length (Z32020253; Yufu Brand, Jiangsu Kangmei Pharmaceutical Co., Ltd, Jiangsu, China), which will be used in the Mild moxibustion group and the Warm-needling acupuncture group.

The unified electro-acupuncture apparatus is the SDZ-IIBb electro-acupuncture apparatus (Huatuo Brand, Jiangsu Suzhou Medical Supplies Factory Co., Ltd, Jiangsu, China), which will be used in the Electro-acupuncture group.

The unified leather measuring tape is 1.5 m × 7.5 mm in size (type 8214; Deli Brand, Deli Group Ltd, Ningbo, China).

The unified treatment time and therapy course: each treatment will last for 30 min. The participants will receive three treatment sessions per week for 4 consecutive weeks.

Acupuncture treatments will be operated by the attending doctors, who have more than 5 years of clinical experience at each centre.

Emergency medication and treatment will be unified. If the participants experience unbearable knee pain during the study, Celebrex capsules (Pfizer Pharmaceuticals Ltd), 0.2 g a time per day orally, can be taken. Drug use, temporary treatment of knee swelling during the study and fluid pumping will also be allowed, but all should be recorded in detail on the CRF.

### Acupuncture group

After routine sterilization of the local skin, a 0.30 mm × 25 mm needle will be inserted into the six acupoints mentioned earlier at a depth of 25–30 mm and manipulated using techniques including lifting, thrusting, twisting and rotating with an even reinforcing–reducing method until De Qi [25] (the sensation of sourness, numbness, heaviness and distention) is achieved. All needles will be removed after 30 min.

### Electro-acupuncture group

The acupoints and acupuncture operation will be the same as those used in the Acupuncture group. The electrode of the electro-acupuncture apparatus will be used in the electro-acupuncture group; it will be connected in Neixiyan (EX-LE4) and Dubi (ST35) using a dilatational wave with a frequency of 2/100 Hz, at a strength that is tolerable to the participants (preferably with the skin around the acupoints shivering mildly without pain), for 30 min.

### Mild moxibustion group

In this group, Liangqiu (ST34), Xuehai (SP10), Yanglingquan (GB 34) and Yinlingquan (SP9) will be needled in the same manner described for the Acupuncture group. At the same time, mild moxibustion will be applied at a distance of 2–3 cm away from the skin of both Neixiyan (EX-LE4) and Dubi (ST35) by two Zhuang for 30 min.

### Warm-needling group

The acupoints, location and acupuncture operation will be the same as those described for the Acupuncture group, except that Neixiyan (EX-LE4) and Dubi (ST35) will be treated with one Zhuang (2 cm) of moxa placed on the top of the needle (0.30 mm × 40 mm type) and burnt at the lower end. Two Zhuang will be applied to each acupoint. During the process of moxibustion, if the participants feel unbearable heat, we will place paper between the skin and the moxa to prevent burns.

### Sham acupuncture group

The sham acupoints will be Sham Liangqiu (ST34), Sham Xuehai (SP10), Sham Neixiyan (EX-LE4), Sham Yanglingquan (GB 34) and Sham Yinlingquan (SP9).

The sham acupuncture group will receive non-meridian and non-acupoint needling. Needling will be performed 1 cm lateral to the above acupoints with a 0.18 mm × 25 mm disposable needle placed superficially at a depth of 1–2 mm for 30 min.

### Celebrex treatment group

Celebrex capsules (Pfizer Pharmaceuticals Ltd) will be given once a day (one-time oral 0.2 g) for 4 weeks. The subjects in this group will return to the clinic every week to receive the tablets for the coming week and report any adverse events related to the treatment.

To improve participants’ adherence to our intervention, we offer free consultation and professional assessment during the trial to all of the participants. In the Celebrex treatment group, we use tablets returned every week. Other than acupuncture treatment, any treatment that may affect the results will be prohibited.

## Outcomes

All subjects will complete a questionnaire during the screening period regarding demographic data, including gender, age, nationality, education level, occupation and marital status, as well as information on past KOA treatment such as the time, duration, method and therapeutic effect. X-ray examination and certain blood test results will also be recorded for analysis before randomization.

Each participant will receive a clinical assessment of primary and secondary outcome measurements at five time points: before treatment (screening period, first visit), after treatment (2nd week and 4th week) and at follow-up (3rd month and 6th month). If both knees of the participants meet the inclusion criteria, the knee with more severe symptoms will be assessed. The schedule of this study is presented in Table 1.

**Table 1:** Schedule of enrolment, treatments and assessments

Study period	Enrolment/baseline	Treatment (weeks)		Follow-up (months)	
Assessment point	1	2	3	4	5
Time	-2 weeks to 0	2 weeks ± 3 days	4 weeks ± 3 days	3 months ± 3 days	6 months ± 3 days
Eligibility screening	X				
Demographic data	X				
Case data	X				
X-ray examination of the knee	X				
Identified diagnosis	X				
Inclusion criteria	X				
Exclusion criteria	X				
Informed consent	X				
Treatment		X	X		
Expectancy questionnaires	X				
Outcome assessment					
(1) VAS	X	X	X	X	X
(2) WOMAC	X	X	X	X	X
(3) PASE	X	X	X	X	X
(4) Knee swelling measurement	X	X	X	X	X
(5) WHO-BREF scale	X	X	X	X	X
Safety assessment		X	X		
Compliance assessment			X		
Other treatment	X				
Adverse events	X				
Data processing	X				
Investigator qualification review	X				

*PASE* Physical Activity Scale of the Elderly, *VAS* visual analogue scale, *WHO-BREF* WHO Quality Of Life-BREF, *WOMAC* Western Ontario and McMaster Universities Osteoarthritis Index

### Primary outcome measurement

#### Visual analogue scale/score

The 0–10 cm VAS will be used to measure pain intensity. The participants will indicate pain intensity on the VAS, with 0 representing no pain and 10 cm representing the worst possible pain. A higher score indicates worse pain. The average pain intensity for the last 3 days will be used. (Time frame: screening period, 2 weeks, 4 weeks, 3 months and 6 months.)

#### WOMAC function subscale

The Western Ontario and McMaster Universities (WOMAC) Osteoarthritis Index [26] function subscale assesses the participant’s physical ability to move around and perform usual activities of daily life. It consists of 17 questions regarding the degree of difficulty experienced due to OA in the studied knee. The WOMAC function subscale score for each question ranges from 0 (no symptoms) to 4 (severe symptoms), with higher scores indicating worse physical function. The overall score ranges from 0 (minimum) to 68 (maximum), with higher scores indicating worse physical function. (Time frame: screening period, 2 weeks, 4 weeks, 3 months and 6 months.)

We will compare the change of VAS score and WOMAC function score between the groups at the stated time points and compare mean scores between groups.

### Secondary outcome measurement

#### WOMAC pain subscale

The WOMAC pain subscale measures the patient’s pain. The scale consists of five items, with each item scored from 0 (no pain) to 4 (extreme pain). The overall score ranges from 0 (minimum) to 20 (maximum), with higher scores indicating worse pain.

#### WOMAC stiffness subscale.

The WOMAC stiffness subscale consists of two questions concerning the levels of stiffness experienced in the studied knee. The WOMAC stiffness subscale score for each question ranges from 0 (minimum) to 4 (maximum), with higher scores indicating worse stiffness. The overall score ranges from 0 (minimum) to 8 (maximum), with higher scores indicating more stiffness.

#### Physical Activity Scale for the Elderly (PASE)

The PASE is a brief (5 min) and easily scored survey designed to assess aged adults’ physical performance. The survey includes questions related to household, leisure and work activities. The total PASE score is computed by multiplying the amount of time spent in each activity (hours/week) or participation (yes/no) in an activity by the empirically derived item weights and summing the results for all activities [27]. (Time frame: screening period, 2 weeks, 4 weeks, 3 months and 6 months.)

#### WHO-BREF 36-Item Form

The WHO-BREF 36-Item Form is a questionnaire consisting of 36 items containing questions regarding eight topics used to assess patients’ quality of life. Lower scores indicate a worse condition. (Time frame: screening period, 2 weeks, 4 weeks, 3 months and 6 months.)

#### Knee swelling measurement

A score from 0 (minimum) to 3 (maximum) indicating swelling severity and the average knee circumference at three levels (superior border, inferior margin and middle) will be determined. (Time frame: screening period, 2 weeks, 4 weeks, 3 months and 6 months.)

#### Expectancy questionnaire

The expectancy questionnaire consists of two questions to assess the credibility and expectations of the patients at baseline, before the first treatment.

We will compare the change of WOMAC pain and stiffness score, PASE score and WHO-BREF score, and knee swelling measurement score and knee circumference at the aforementioned time points between groups and compare mean scores between the six groups.

### Incidence of adverse events

All adverse events occurring during the trial—such as fainting, sweating or dizziness; skin burns during acupuncture and moxibustion treatment; or bleeding, local haematoma and other abnormal feelings after acupuncture treatment—will be recorded on the CRF. The researcher will record all details, such as the date, time and degree of occurrence related to the treatment at each visit, and will provide proper medical advice and care. Severe adverse events will be reported to the principal investigator and the ethics board immediately, and proper measures will be taken in a timely manner.

## Quality control and data collection

The study protocol is based on considerable document research and trial tests, and has been reviewed by several well-known acupuncture and orthopaedics experts, statisticians and computer engineers. Before the first patients are enrolled, all staff will be required to attend a training session about the overall study protocol, the qualification standards of each unified treatment operation, the investigator’s brochure and completion of the CRF form. During the study, the Clinical Evaluation and Analysis Centre of Zhejiang Provincial Hospital of TCM is responsible for generating the allocation sequence, for quality control and for censors making regular visits (once a month) to monitor for protocol violations, the recruitment rate, AEs and participant compliance. This clinical trial is independent from sponsors and competing interests. The supervisory team will check the case reports, treatment operation and data entry quality at each centre twice a month. During the trial, the principle investigator and other research team members will meet once a month to discuss the project’s progress in terms of enrolment, withdrawal and case loss, treatment compliance and adverse events as well as methods for improving the participants’ compliance. All of the data will be collected in the CRF and double entered into the EDC system electronically by independent researchers at the set time. Drop-outs and withdrawals from the trial will be recorded throughout the intervention and follow-up periods.

## Sample size and statistical analysis

### Sample size

The sample size was calculated by PASS version 11.0. According to document analysis and the pilot trial, the WOMAC joint function scores changed by 13.0 ± 5.6 after treatment in a positive drug control group [28], while the needling group changed by 8.8 ± 3.7 [29], the EA group changed by 8.9 ± 4.0 (pilot trial), the Moxibustion group changed by 10 ± 4.7 (pilot trial), the Warm-needling group changed by 12.0 ± 6.1 [30] and the Sham acupuncture group changed by 5.8 ± 3.0 [29]. With the study power maintained at 0.9, an α level of 0.05 and a 1:1:1:1:1:1 ratio, the sample size required is at least 42 participants per group. With the consideration of a 20% drop-out rate, at least 53 patients are required in each group. In view of the equality of distribution of all cases in the six centres, there will be a total of 360 cases with 60 patients in each group. The following formula was used:

$$ n={\psi}^2\left[\sum \limits_{i=1}^k{s}_i^2/k\right]/\left[\sum \limits_{i=1}^k{\left({\overline{X}}_i-\overline{X}\right)}^2/\left(k-1\right)\right]. $$

### Statistical analysis

The statistical analysis will be performed using SAS for Microsoft Windows version 9.3 by a single statistician from the Clinical Evaluation and Analysis Centre of Zhejiang Provincial Hospital of TCM. Comparisons of categorical data among groups will be tested by the chi-squared test or Fisher’s exact test. Changes in the scores from baseline within treatment groups will be assessed by the paired *t* test or analysis of covariance (ANCOVA). The efficacy of the six centres will be tested by the Cochran–Mantel–Haensel (CMH) method for count data and covariance analysis for measurement data. A safety analysis will be performed by analysing the frequency of adverse events related to the treatment reported by researchers at every visit. The missing values will be analysed by the last observation carried forward method and then analysed using the intention-to-treat analysis for primary outcome measurements. The available case analysis principle will be used when dealing with missing values for secondary outcome measurements, general characteristics and safety analyses. *p* < 0.05 (α = 0.05) is considered to indicate statistical significance.

## Discussion

Osteoarthritis is thought to be the most prevalent chronic joint disease. The incidence of osteoarthritis is rising because of the ageing population, and little is known about the complex mechanisms of disability accumulation in KOA patients [31, 32]. Acupuncture is a popular non-pharmacological modality for treating a variety of conditions, and may result in reduced opioid use [33]. The different kinds of acupuncture treatment for KOA studied in this trial are widely used in the clinic, and sham acupuncture is often used as a control or placebo in many high-quality clinical studies. However, few clinical trials have confirmed the effectiveness and safety of acupuncture therapies for KOA. The evidence is scarce, as is information regarding the effect size [34]. Acupuncture has not been recognized as a standard treatment for KOA, and it has been excluded from Western medicine. In previous studies, very few reported clinical trials have used valid medicine and sham acupuncture as control groups at the same time. Large-sample multicentre RCTs are still lacking.

Therefore, we will strive to conduct a rigorous, parallel, single-blinded, multicentre clinical trial with high methodological quality using different controls and conducted by well-qualified acupuncture doctors with rich work experience. The outcomes of the VAS, WOMAC, PASE, quality of life and knee swelling measurement are well-suited for evaluating the clinical efficacy of KOA. Pain, physical function and global patient assessment are presumed to be gold standards for self-reported measures in KOA trials [35]. This study will provide convincing data on the efficacy of different forms of acupuncture and moxibustion for KOA through a follow-up period of 6 months after completion of the treatment.

The protocol design incorporates the recommendations of the Osteoarthritis Research Society International (OARSI) and Standard Protocol Items: Recommendations for Interventional Trials [20, 36, 37]. However, the recommended blinding method will be difficult to apply in this trial. Only a single-blind method can be applied in this study because most patients will have had acupuncture treatment experience before this trial. To reduce performance bias, the assessor, data recorder, operator and statistician will be blinded to group allocation. In the sham acupuncture group, we will needle superficially at non-acupoints belonging to non-meridians to assist in the implementation of the blinding method according to the principle of providing contrast.

In conclusion, this multicentre, parallel, single-blinded RCT will investigate the efficacy and safety of different kinds of acupuncture treatments for KOA, provide convincing evidence, assess the feasibility and relevance of traditional acupuncture and sham acupuncture study designs, improve the standardization of acupuncture and moxibustion for KOA, and provide a clinical foundation for future large-scale pluralistic clinical trials.

### Trial status

This trial is currently recruiting participants. Protocol version 3.0 is dated 17 April 2019. The recruitment procedure began on July 2018. The authors expect to complete this procedure on 31 December 2021.

## Supplementary information

**Additional file 1.** Reporting checklist for the protocol of a clinical trial.

## Abbreviations

ACR: American College of Rheumatology
ANCOVA: Analysis of covariance
CMH: Cochran–Mantel–Haensel
KL: Kellgren–Lawrence
KOA: Knee osteoarthritis
OARSI: Osteoarthritis Research Society International
PASE: Physical Activity Scale of the Elderly
VAS: Visual analogue scale
WHO: World Health Organization
WHOQOL-BREF: WHO Quality Of Life-BREF
WOMAC: Western Ontario and McMaster Universities Osteoarthritis Index
